# Bovine Herpesvirus 1 Counteracts Immune Responses and Immune-Surveillance to Enhance Pathogenesis and Virus Transmission

**DOI:** 10.3389/fimmu.2019.01008

**Published:** 2019-05-07

**Authors:** Clinton Jones

**Affiliations:** Department of Veterinary Pathobiology, Center for Veterinary Health Sciences, Oklahoma State University, Stillwater, OK, United States

**Keywords:** bovine herpesvirus 1 (BoHV-1), immune evasion, VP8, infected cells protein 0 (bICP0), abortion, bovine respiratory disease complex

## Abstract

Infection of cattle by bovine herpesvirus 1 (BoHV-1) can culminate in upper respiratory tract disorders, conjunctivitis, or genital disorders. Infection also consistently leads to transient immune-suppression. BoHV-1 is the number one infectious agent in cattle that is associated with abortions in cattle. BoHV-1, as other α-herpesvirinae subfamily members, establishes latency in sensory neurons. Stressful stimuli, mimicked by the synthetic corticosteroid dexamethasone, consistently induce reactivation from latency in latently infected calves and rabbits. Increased corticosteroid levels due to stress have a two-pronged effect on reactivation from latency by: (1) directly stimulating viral gene expression and replication, and (2) impairing antiviral immune responses, thus enhancing virus spread and transmission. BoHV-1 encodes several proteins, bICP0, bICP27, gG, UL49.5, and VP8, which interfere with key antiviral innate immune responses in the absence of other viral genes. Furthermore, the ability of BoHV-1 to infect lymphocytes and induce apoptosis, in particular CD4+ T cells, has negative impacts on immune responses during acute infection. BoHV-1 induced immune-suppression can initiate the poly-microbial disorder known as bovine respiratory disease complex, which costs the US cattle industry more than one billion dollars annually. Furthermore, interfering with antiviral responses may promote viral spread to ovaries and the developing fetus, thus enhancing reproductive issues associated with BoHV-1 infection of cows or pregnant cows. The focus of this review is to describe the known mechanisms, direct and indirect, by which BoHV-1 interferes with antiviral immune responses during the course of infection.

## BoHV-1 is an Important Viral Pathogen

Bovine herpesvirus 1 (BoHV-1) is an α-herpesvirinae subfamily member that causes significant economical losses to the cattle industry (1). Three well-defined subtypes exist, BoHV-1.1, BoHV-1.2a, and BoHV-1.2b (2b) (2). Subtype 1 virus isolates are prevalent in Europe, North America, and South America: these subtypes are frequently detected in cattle suffering from infectious bovine rhinotracheitis (IBR) and the respiratory tract of aborted fetuses. Subtype 2a strains are prevalent in Brazil and are associated with respiratory and genital tract infections, including IBR, infectious pustular vulvovaginitis (IPV), balanopostitis (IPV), and abortions (3). Subtype 2b strains, which are frequently isolated in Australia or Europe (4), are associated with respiratory disease and IPV/IPB, but not abortion (3, 5). The seroprevelance of BoHV-1 ranges from 14 to 90% depending on the age of cattle and geographical location (6, 7). Serological testing and removal of infected animals has eliminated BoHV-1 from Denmark, Switzerland, and Austria (8).

BoHV-1 is the most frequently diagnosed cause of viral abortion in North American cattle (9). Exposure of a susceptible herd to BoHV-1 can result in abortion storms ranging from 25 to 60% of cows undergoing abortion. Commercially available modified live vaccines also induce abortions in pregnant cows. Furthermore, several studies concluded that naïve heifers vaccinated with an inactivated BoHV-1 vaccine are more likely to have a normal estrous cycle and significantly higher pregnancy rates relative to heifers vaccinated with a modified live (MLV) vaccine (9–13).

The incubation period for the genital forms of BoHV-1 is 2–6 day and initial clinical signs are frequent urination and a mild vaginal infection (14). It is also common to observe swollen vulva or small papules followed by erosions and ulcers on the mucosal surface. In bulls, similar lesions occur on the penis and prepuce. If secondary bacterial infections occur, inflammation of the uterus and transient infertility with purulent vaginal discharge occurs for several weeks. BoHV-1 infection, virulent field strains or modified live vaccines, of sero-negative heifers can target the ovary and corpus luteum during estrus and early in gestation (9).

Bovine respiratory disease complex (BRDC), a poly-microbial disease initiated by stress and/or virus infection, is the most economically important disease that affects beef and dairy cattle. Annual BRDC losses in the U.S. are ~$1 billion (15–18). A gram negative bacterium, *Mannheimia haemolytica (MH)*, exists in the upper respiratory tract of healthy ruminants (19, 20). Following stressful stimuli or co-infections with other viruses (21), this commensal relationship is disrupted and *MH* becomes the predominant organism that causes life threatening bronchopneumonia in many BRDC cases (22–25). BoHV-1 infection frequently causes upper respiratory tract disease (26, 27), high fever, conjunctivitis, and erodes mucosal surfaces of the upper respiratory tract. Consequently, colonization of *MH* occurs in the lower respiratory tract (22, 23, 25), thus enhancing interactions between the *MH* leukotoxin, bovine peripheral blood mononuclear cells, and neutrophils (28, 29). Co-infection of calves with BoHV-1 and *MH* consistently leads to pneumonia (30). Finally, a BoHV-1 protein that is required for virus entry was identified as a significant BRDC susceptibility gene in Holsteins (31) confirming BoHV-1 is an important BRDC cofactor.

## The BoHV-1 Latency-Reactivation Cycle is Important for Virus Transmission

### Acute Infection Leads to High Levels of Virus Shedding

Acute BoHV-1 infection of cattle is initiated on mucosal surfaces and results in high levels of programmed cell death (32, 33). Acute infection leads to high levels of virus production and secretion in ocular, oral, nasal, or genital cavities for 7–10 days after infection. BoHV-1 gene expression during productive infection is operationally divided into three distinct phases: immediate early (IE), early (E), or late (L) (32, 33). IE gene expression is stimulated by VP16, a tegument protein (34, 35). Thus, IE mRNA expression does not require *de novo* protein synthesis. Two IE transcription units exist: IE transcription unit 1 (IEtu1) and IEtu2. IE transcription unit 1 (IEtu1) encodes two transcriptional regulatory proteins, bICP0 and bICP4, because a single IE transcript is differentially spliced and then translated into bICP0 or bICP4 (36–38). The bICP0 protein is also translated from an E mRNA (E2.6) because a separate E promoter drives expression of the bICP0 E transcript (36–39). The bICP0 protein has similar properties as HSV-1 encoded ICP0 (40), including a RING finger that is crucial for stimulating viral promoters and productive infection (41, 42). bICP4 is likely to possess similar functions as the HSV-1 encoded ICP4. bICP4 autoregulates the IEtu1 promoter, but activates the bICP0 E promoter.

E gene expression requires *de novo* protein expression, including bICP0 and bICP4, which transactivate E viral promoters. In general, the E proteins encode proteins that promote DNA synthesis. Example of early viral proteins include the DNA polymerase, thymidine kinase, small and large subunits of the ribonucleotide reductase, dUTPase, and origin binding protein. In general, the E proteins are non-structural.

The L genes are divided into two classes: Gamma-1 and Gamma-2 genes. Transcription of Gamma-1 genes requires *de novo* protein synthesis, including bICP0 and bICP4, but does not require viral DNA replication. Transcription of Gamma-2 genes requires *de novo* protein synthesis, including bICP0 and bICP4, and abundant expression requires viral DNA replication. In general, L proteins encode structural proteins and their synthesis culminates in virion assembly and release.

### Summary of Latency-Reactivation Cycle

Viral particles enter the peripheral nervous system via cell-cell spread. If infection is initiated within the oral, nasal, or ocular cavity, the primary site for latency is sensory neurons in trigeminal ganglia (TG). Viral gene expression (43) and infectious virus (44) are detected in TG from 2 to 6 days after infection. Lytic gene expression is then extinguished, and surviving infected neurons harbor viral genomes (establishment of latency).

Abundant expression of the viral encoded latency related (LR) gene occurs in latently infected neurons, but infectious virus is not readily detected (maintenance of latency) (32, 33, 45–48). LR-RNA overlaps the bICP0 gene (49, 50), has two open reading frames (ORF1 and ORF2), two reading frames lacking an initiating ATG, and encodes two micro-RNAs. A LR mutant virus strain with three stop codons at the N-terminus of ORF2 has reduced virus shedding from the eye, TG, or tonsils of infected calves (44, 51, 52). LR-encoded proteins are expressed late during productive infection when infected with wild-type (wt) or LR-rescued virus, but have reduced or no expression after infection with the LR mutant virus (53, 54). Wt BoHV-1, but not the LR mutant virus, reactivates from latency (44). The anti-apoptosis activity of ORF2 (41, 55–57) and the micro-RNAs, which interfere with bICP0 expression (58) regulate the latency-reactivation cycle.

The synthetic corticosteroid dexamethasone (DEX) initiates reactivation from latency in latently infected calves or rabbits 100% of the time (27, 32, 33, 44, 47, 59). Within 6 h after latently infected calves are treated with DEX, viral regulatory proteins (ICP0 and VP16) (60, 61) and lytic cycle viral RNA expression are detected in TG neurons (62, 63). Within 3 h after DEX treatment, 11 cellular genes are induced more than 10-fold in TG (64). Pentraxin 3, a regulator of innate immunity and neuro-degeneration, is stimulated more than 30-fold at 3 or 6 h after DEX treatment. Two transcription factors, promyelocytic leukemia zinc finger (PLZF) and Slug are induced more than 15-fold 3 h after DEX treatment, which can enhance productive infection. Additional DEX induced transcription factors, SPDEF (Sam-pointed domain containing Ets transcription factor), Krüppel-like transcription factor 15 (KLF15), KLF4, KLF6, and GATA6, stimulate productive infection and certain key viral promoters. The finding that four KLF family members are stimulated during DEX induced reactivation from latency is intriguing because KLF family members resemble the Sp1 transcription factor family and both family of transcription factors interact with GC rich motifs, reviewed in Bieker (65) and Kaczynski et al. (66). The BoHV-1 genome is GC rich and many viral promoters contain Sp1 consensus binding sites and other GC rich motifs suggesting specific KLF transcription factors bind to viral sequences and stimulate viral transcription during early stages of reactivation from latency.

The IEtu1 promoter that drives bICP0 and bICP4 expression is stimulated by DEX and contains two consensus GR binding sites that are bound by the activated GR (67, 68). The GR and KLF15 are frequently expressed in the same TG neuron during reactivation and cooperatively stimulate productive infection and IEtu1 promoter activity. A host cellular factor 1 (HCF-1), which forms a complex with VP16 and Oct1 to bind to the IE enhancer core via the TAATGARAT motif, is important for GR mediated activation of the IEtu1 promoter suggesting glucocorticoid induction of viral reactivation may proceed via an HCF-1-GR mechanism in the absence of the viral IE activator VP16 (69). Stress-mediated activation of key viral promoters is predicted to be a very early event during reactivation from latency; then viral transactivators activate all other viral genes and virus production occurs. Hence, stress has a two-pronged effect on reactivation from latency by directly activating viral gene expression and indirectly enhancing viral spread via immunosuppression (70–72).

## Immune Response to BoHV-1 Following Acute Infection

Cattle acutely infected with BoHV-1 develop an innate immune response (73–76); however, efficient virus replication and spread occurs. For example, virus neutralizing antibodies are detected after acute infection that recognize envelope glycoproteins, including gB, gC, gD, and gH (77, 78). Cytotoxic T cell responses to viral glycoproteins occur in cattle following infection (79–81). Infection of cultured cells also induces inflammasome formation (82), consistent with inflammation in the nasal cavity and upper respiratory tract during acute infection.

**Figure 1:**
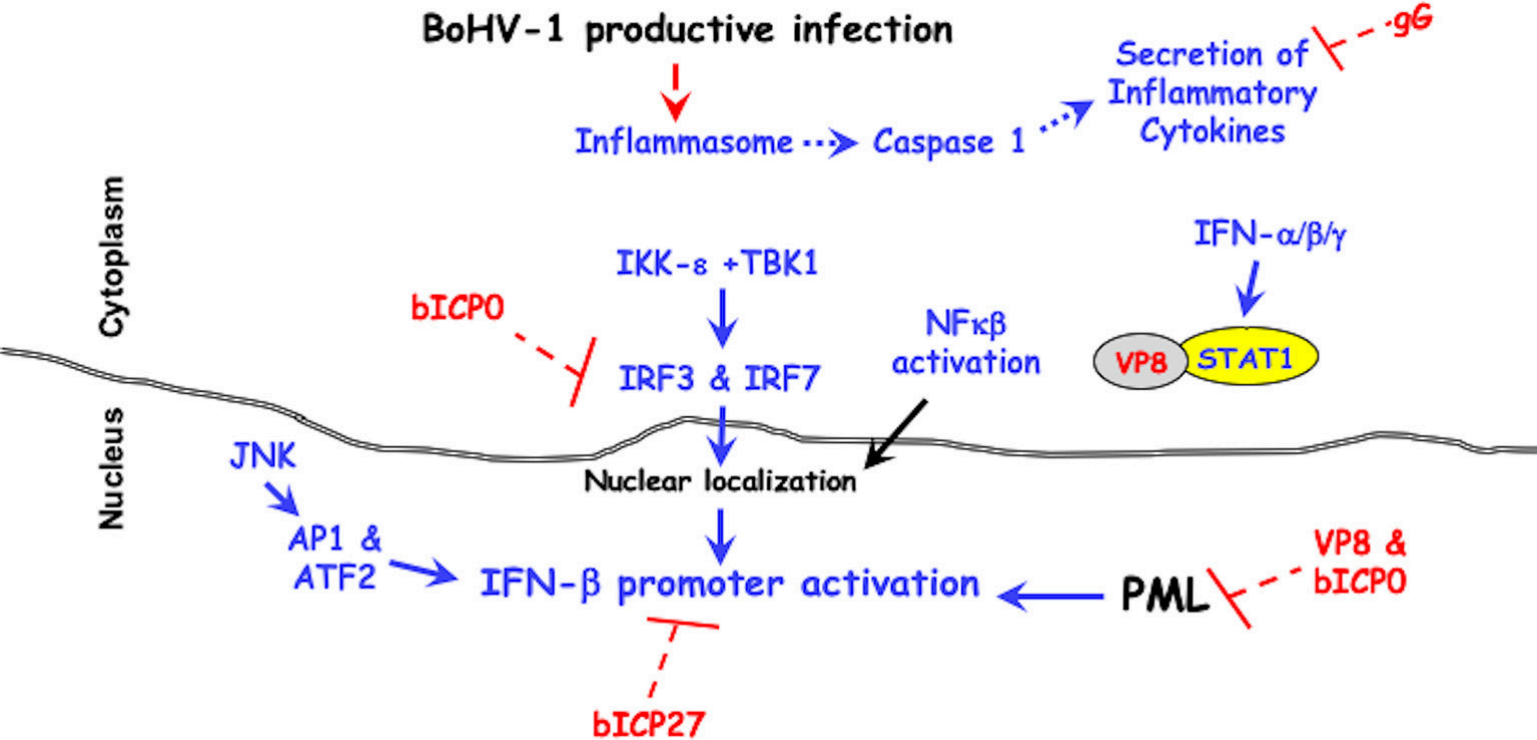
BoHV-1 encoded immune-evasion genes that promote productive infection. Cellular mechanisms leading to innate immune antiviral signaling pathways are denoted in blue. Red lettering denotes viral genes that counteract antiviral signaling pathways. It is well-established that two protein kinases (IKK-ε +TBK1) activate the transcription factors (IRF3 and IRF7), which are required for activating the IFN-β promoter (96–98). The JNK protein kinase (c-Jun N-terminal kinases) activates the AP1 (activating protein 1) and ATF2 (activating transcription factor 2), which are also required for activating the IFN-β promoter (96–98). For further details, see the text.

Although the host immune response clears virus after acute infection, viral infection impairs immune-recognition on several levels impairs: (1) cell-mediated immunity (83–86), (2) CD8+ T cell recognition of infected cells (68, 87–89), (3) CD4+ T cell functions because BoHV-1 infect these cells and rapidly inducing apoptosis after viral entry (90, 91), and (4) interferon responses (92–95). The known viral genes that antagonize immune responses are discussed below (see Figure 1 for a schematic that summarizes how viral genes impair immune responses).

## Viral Proteins Interfere With Innate Immune Responses and Immune-Surveillance

The amino-terminus of the bICP0 protein contains transcriptional activation domains, a nuclear localization signal (NLS) necessary for efficient transcriptional activation (99), and a C_3_HC_4_ zinc RING finger that is conserved in all ICP0 proteins (100, 101). Point mutations within the C_3_HC_4_ zinc RING finger domain of bICP0 interfere with transactivation of a simple viral promoter (99), stimulation of productive infection (41, 102), and reduces IFN-β promoter activity (92–95). bICP0 co-localizes with and disrupts the anti-viral promyelocytic leukemia (PML) protein-containing nuclear domains (41, 101). PML bodies are comprised of numerous proteins, which regulate the cell cycle, apoptosis, senescence, stress, DNA damage, and innate immune responses (103). Many DNA viruses reorganize or dissolve PML bodies, thus increasing viral replication. Interferon treatment increases components of PML bodies, Sp100, and PML for example (104, 105) and PML bodies increase beta-interferon (IFN-β) expression (106).

bICP0 inhibits IFN-β promoter activity in transient transfection studies (92, 94) by reducing IRF3 (interferon regulatory factor 3) protein levels. The RING finger of bICP0 (107) is an E3 ubiquitin ligase suggesting it mediates IRF3 degradation in a proteasome dependent manner. bICP0 also interacts with IRF7 and impairs activation of IFN-β promoter activity, but does not reduce IRF7 protein levels (94). IRF3 and IRF7 are transcription factors that stimulate IFN-β promoter activity (96–98). IRF3 directly binds several consensus DNA binding sites, including an ISRE (IFN response elements), and can activate IFN-stimulated promoters in the absence of IFN (108, 109). A recent study concluded PML regulates intrinsic and innate immune responses to HSV-1 infection, which is ablated by ICP0 (110). The ability of bICP0 to reduce IFN-β promoter activity correlates with IRF3 degradation, IRF7 interactions, and dissolving PML bodies.

The BoHV-1 bICP27 protein is expressed from an early promoter and based on similarity with the HSV-1 ICP27 is expected to shuttle RNA from the nucleus to the cytoplasm and regulate transcription (111). HSV-1 encoded ICP27 regulates IFN expression (112) by interfering with activation of the stimulator of interferon genes (STING) by tank binding protein kinase 1 (TBK1) (113). Interestingly, bICP27 reduces bovine IFN-β1 and IFN-β3 promoter activity in transfected cells (114). *Bos Taurus* encodes three functional IFN-β genes; all have anti-viral activity but each gene contains a unique promoter (115, 116).

Glycoprotein G (gG) promotes cell to cell spread (117) and maintains adherence of infected cells (118). gG is a unique viral glycoprotein because it can exist in three isoforms: a full-length membrane-bound form, a smaller membrane-bound form, and a secreted form. gG interferes with chemokine binding to their specific receptors and glycosaminoglycans (119). Although it is not known what role gG plays during acute infection of calves, the ability of chemokines to control the migratory patterns and positioning of immune cells (120) would likely be altered by gG.

The BoHV-1 UL49.5 ORF, also known as glycoprotein N (gN), is a 96 amino acid protein (121). The BoHV-1 and pseudorabies virus UL49.5 proteins interfere with processing of the transporter-associated antigen processing (TAP)-mediated transport of cytosolic peptides into the endoplasmic reticulum because UL49.5 renders the TAP complex susceptible to proteolytic degradation (122, 123). Peptide transport by TAP is crucial for MHC class I antigen presentation and recognition of infected cells by CD8^+^ T cells (122, 124–126). Infection of calves with a UL49.5 BoHV-1 mutant leads to increased levels of virus neutralizing antibody and cellular immune responses when compared to the parental wild-type virus (127).

VP8, the most abundant tegument protein in the virion, enhances growth in cultured cells and is required for pathogenesis in calves (128). VP8 interacts with DDB1 (DNA damaging-binding protein 1) that is associated with a E3 ubiquitin ligase complex (129), and remodels PML nuclear bodies (130). Recent studies demonstrated VP8 interacts with STAT1 (Signal transducer and activator of transcription 1) and prevents STAT1 from entering the nucleus (131). Stat1 is bound to the IFN-γ receptor and upon IFN-γ binding to its receptor (Jak1 and Jak2) phosphorylates specific tyrosine residues on STAT1. STAT1 subsequently enters the nucleus and stimulates GAS (IFN-γ activated sequences) setting off a second wave of IFN-γ (132). Following IFN-α or IFN-β stimulation, STAT1 forms a heterodimer with STAT2 and this heterodimer binds an ISRE element and activates transcription (133). VP8 also interferes with IFN-β signaling activity by reducing an interferon sensitive response element (ISRE) responsive promoter in transfected or infected cells. Thus, VP8 is a potent IFN antagonist that can interfere with host innate immune responses in the absence of *de novo* viral protein synthesis.

## Conclusions/Discussion

BoHV-1 is a very successful pathogen because it encodes several genes that impair intrinsic and innate immune responses throughout productive infection (see Figure 1). VP8 is likely the initial anti-viral protein that impairs antiviral IFN responses because high levels of VP8 are present in the tegument of incoming viral particles. bICP0, which is encoded by the IEtu1 promoter, would be an early interferon antagonist. bICP27 via unknown mechanisms interferes with IFN-β promoter activation. Three late proteins (gG, UL49.5, and VP8) would further antagonize immune-recognition. In summary, the presence of viral proteins in the virion and expression of viral proteins throughout productive infection allows for high levels of virus production during acute infection and reactivation from latency in cattle.

## Author Contributions

The author confirms being the sole contributor of this work and has approved it for publication.

### Conflict of Interest Statement

The author declares that the research was conducted in the absence of any commercial or financial relationships that could be construed as a potential conflict of interest.
